# Infective Endocarditis Epidemiology Over Five Decades: A Systematic Review

**DOI:** 10.1371/journal.pone.0082665

**Published:** 2013-12-09

**Authors:** Leandro Slipczuk, J. Nicolas Codolosa, Carlos D. Davila, Abel Romero-Corral, Jeong Yun, Gregg S. Pressman, Vincent M. Figueredo

**Affiliations:** 1 Department of Medicine, Einstein Medical Center, Philadelphia, Pennsylvania, United States of America; 2 Heart Institute, Cedars-Sinai Medical Center, Los Angeles, California, United States of America; 3 Einstein Institute for Heart and Vascular Health, Einstein Medical Center, Philadelphia, Pennsylvania, United States of America; 4 Pulmonary and Critical Care Medicine Division, Brigham and Women’s Hospital, Boston, Massachusetts, United States of America; 5 Jefferson Medical College, Philadelphia, Pennsylvania, United States of America; University of Iowa Carver College of Medicine, United States of America

## Abstract

**Aims:**

To Assess changes in infective endocarditis (IE) epidemiology over the last 5 decades.

**Methods and Results:**

We searched the published literature using PubMed, MEDLINE, and EMBASE from inception until December 2011.

**Data From:**

Einstein Medical Center, Philadelphia, PA were also included. Criteria for inclusion in this systematic review included studies with reported IE microbiology, IE definition, description of population studied, and time frame. Two authors independently extracted data and assessed manuscript quality. One hundred sixty studies (27,083 patients) met inclusion criteria. Among hospital-based studies (n=142; 23,606 patients) staphylococcal IE percentage increased over time, with coagulase-negative staphylococcus (CNS) increasing over each of the last 5 decades (p<0.001) and *Staphylococcus aureus* (SA) in the last decade (21% to 30%; p<0.05). Streptococcus viridans (SV) and culture negative (CN) IE frequency decreased over time (p<0.001), while enterococcal IE increased in the last decade (p<0.01). Patient age and male predominance increased over time as well. In subgroup analysis, SA frequency increased in North America, but not the rest of the world. This was due, in part, to an increase in intravenous drug abuse IE in North America (p<0.001). Among population-based studies (n=18; 3,477 patients) no significant changes were found.

**Conclusion:**

Important changes occurred in IE epidemiology over the last half-century, especially in the last decade. Staphylococcal and enterococcal IE percentage increased while SV and CN IE decreased. Moreover, mean age at diagnosis increased together with male:female ratio. These changes should be considered at the time of decision-making in treatment of and prophylaxis for IE.

## Introduction

Infective endocarditis (IE) extols a high cost for society worldwide, with a US incidence of 10,000 to 15,000 cases each year[1]. IE is associated with prolonged hospitalization, can require surgery[2], and impairs quality of life[3]. IE was initially described in 1885 by Osler[4] as a disease of patients with pre-existing valvular abnormalities. Since then, notable improvements in IE diagnosis and treatment have been made. However, in-hospital mortality is still close to 20 percent[5,6]. 

Risk factors for IE have changed over time. There have been widespread changes in health-care delivery in the last five decades, which have impacted the clinical spectrum of IE. These include the use of intracardiac devices[7], prosthetic valves[8], hemodialysis[9], and an increase in the elderly population[10]. Furthermore, changes in antibiotics have led to alterations in patterns of infection and bacterial resistance in both the US[11,12] and Europe[13]. 

In recent decades, several studies have noted an increase in the proportion of IE caused by staphylococcal species[14,15]. However, others have not[16]. A systematic review of population-based studies including 15 studies and 2,371 cases found no significant changes in the causative organism over time[17]. However, significant limitations were present in this study, including a low power to detect changes; nor did it cover the last decade. Moreover, to the best of our knowledge, there are no systematic reviews of hospital based studies.

Proper understanding of IE epidemiology is paramount, as different organisms produce varied complications and may require different treatment and prophylaxis[18]. The objective of this research was to assess whether there have been changes in IE epidemiology globally over the last half century. Towards this end, we performed a systematic review of both population and hospital-based studies. 

## Methods

### Data Sources and Searches

We searched, with no language restrictions, PubMed, OVID/MEDLINE and EMBASE electronic databases from their inception to December 2011, for studies reporting infective endocarditis microbiology. We used the term ‘infective endocarditis’ for the Mesh keyword. Date last search performed was December 1^st^, 2011. We supplemented the search with references from articles reviewed and correspondence with other researchers, including experts in the field. When a reference was deemed potentially suitable for inclusion, a full-text copy was obtained and reviewed according to predefined criteria (listed below). We followed the PRISMA guidelines for systematic reviews. 

### Study Selection and Data Collection

Prospective and nonprospective studies reporting the frequency distribution of infective endocarditis in the last five decades were included in this systematic review. Two investigators had the protocol for study selection (LS and CD) and independently assessed the studies for eligibility. Inclusion criteria were: (1) a clear definition of the population studied; (2) a clear definition of the time period; (3) a clear definition of infective endocarditis; and (4) a clear description of the frequency distribution of the microbiology encountered. In order to avoid bias, we excluded studies limited to specific populations (e.g. HIV or intravenous drug users). When there was difference of opinion, a third investigator (NC) resolved the disagreement. The authors are fluent in English, Spanish, Portuguese, and Italian; papers in other languages were translated by collaborating physicians who were native speakers. When two studies reported data from the same cohort and time frame, only the most complete one was included. If a study reported data for various time frames, data were analyzed separately for each decade. Kappa (k) for inclusion was calculated from a sample of 10 randomly selected papers. Results were compared and inconsistencies were resolved by consensus. Dr. Andrew Wang from Duke University was consulted for reviewing the list of included studies for completeness.

For each study included, the following information was extracted: first author’s last name, journal, year of publication, IE definition used, certainty (possible IE vs. definite IE), countries, time-frame, multi-center vs. single center, sample size, age, gender, mortality, intravenous drug abuse (IVDA), intracardiac device or prosthetic valve, *Staphylococcus aureus* (SA), coagulase-negative staphylococcus (CNS), enterococci, Streptococcus viridans (SV) and culture negative (CN) IE. 

We also included patients from Einstein Medical Center (EMC), a tertiary 440-bed city hospital in Philadelphia, PA, US; for the years 2000 - 2010. Data were retrospectively extracted per ICD code and then included only if patients met ‘definite’ or ‘possible’ modified Duke criteria[19]. Clinical characteristics were extracted for each patient as mentioned above. This data was added as one more study to the last decade. Sensitivity analyses showed that subtracting this data from the rest did not modify results significance. 

### Quality assessment

Two reviewers (LS and CD) independently assessed the quality of the manuscripts using the approaches recommended by Khan and colleagues[20] and Stroup and colleagues[21] for cohort studies. The main criteria were: (1) prospective study, (2) Duke or Von Reyn definition of IE, (3) definite IE, and (4) number of patients above 40. Quality was assigned as A, or excellent, with 4 points, B or good, with 2-3 points, and C, or suboptimal, with 0-1 points. Data was weighted for quality for SA and SV without affecting results statistical significance. 

### Statistical analysis

Data were extracted independently by two researchers (LS and CD) and collected on an Excel spreadsheet. Data were allocated to a decade according to the midpoint date for the time frame studied. For example if the time frame was 1979 to 1983, data was assigned to the 80s. Results are shown as mean +/- SD, percentages for each decade, and the 95% CI. Main variables studied were the frequency distribution of pathogens, patient age and gender, and in-hospital mortality. ANOVA and Chi^2^ test were used to compare studies by decades. Each group was compared to the rest using paired Students t-tests. Samples were weighted for size. Results from our own hospital were included in the last decade and weighted for size. Sensitivity analysis showed that subtracting this data did not affect results statistic significance. Moreover subtracting the biggest study by Murdoch et al. from the International Collaboration on Endocarditis did not affect statistical significance of results. Sub-analyses were performed for continent and IVDA. Correlation between IVDA and SA was performed using Spearman correlation test. Hospital-based studies and population-based studies were included. As both types of studies may be prone to bias (hospital-based studies to referral bias[22] and population-based studies to selection bias) hospital-based and population-based studies were analyzed separately. Sensitivity analyses were performed for size, single vs multi-center and quality without affecting significance of results. A p<0.05 was considered statistically significant. Analyses were performed using JMP version 10.0 (SAS Institute, Cary, NC, US). 

Data were presented in a graph as mean (in green, centerline of diamond) and variance (size of diamond) for each variable studied in each decade, with standard deviations (blue). Each dot in a column represents a particular study percentage. N below decades represents total number of patients in each decade. Every patient included in each study was diagnosed with IE as described above. 

## Results

**Figure 1:**
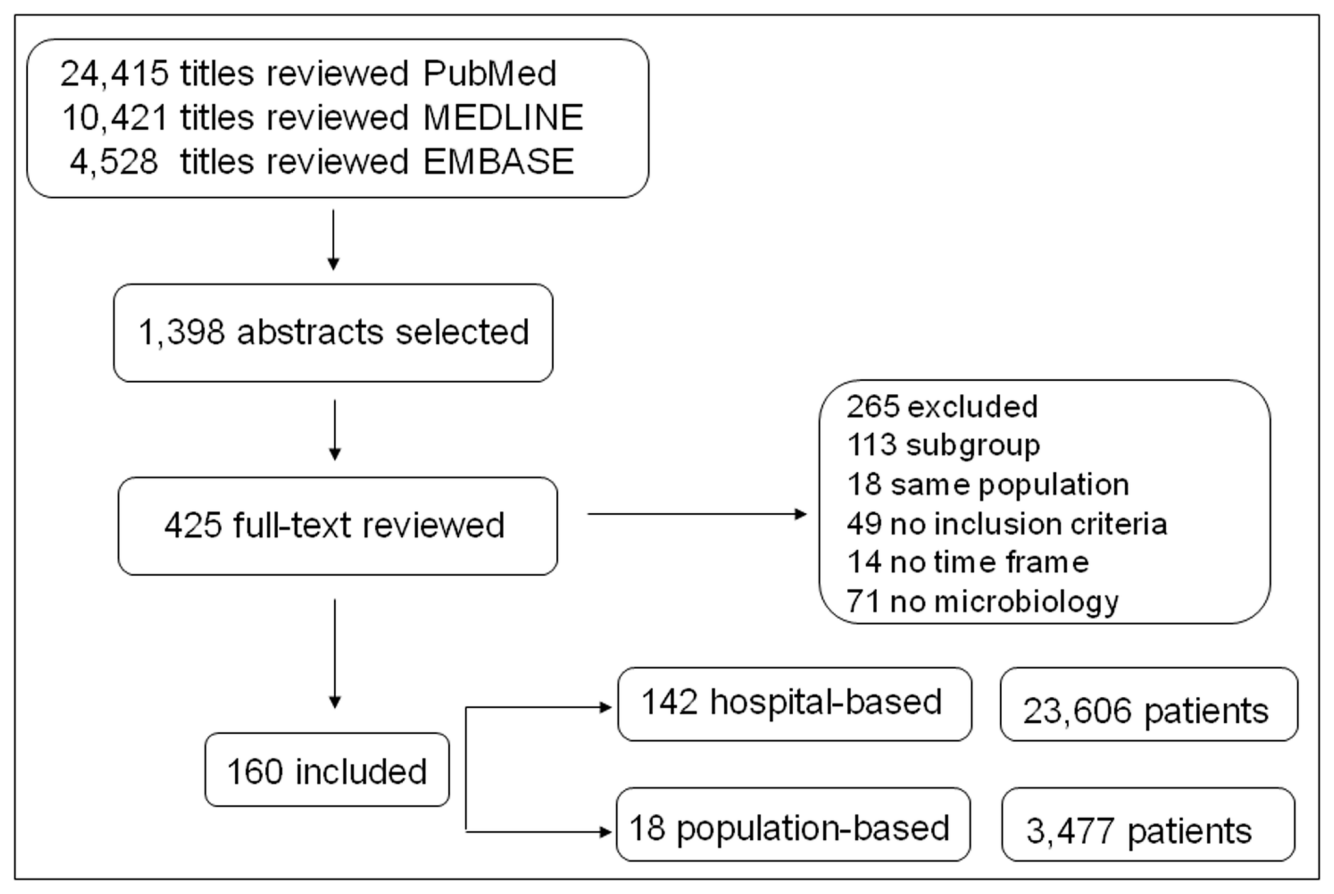
Flowchart of the selection process.

Candidate studies included 24,415 articles identified in PubMed, 10,421 in Medline, and 4,528 in EMBASE; one hundred sixty studies met all inclusion criteria (see flow diagram in Figure 1). Of these, 142 were hospital-based, including a total of 23,606 patients (Table 1), and 18 were population-based, including a total of 3,477 patients (Table 2). Investigators (LS and CD) were in agreement on which articles were to be included (k=1).

**Table 1:** Characteristics of hospital-based studies included.

**Author**	**N**	**De**	**Definition**	**Cert**	**Age**	**Male**	**SA**	**SV**	**CNS**	**Ent**	**CN**	**Mort**	**IVDA**	**Prosthetic**	**Year**	**Country**	**Quality**
Agüero[33]	25	R	Pelletier	NR	NR	NR	12	40			12				77-80	Chile	C
AIPEI[34]	390	P	Duke	Def	60	71	23	16	6	7	5	17	6	16	99	France	A
Ako[35]	69	R	Duke	Pos	55	72	15	38		10	25	19	0	33	90-99	Tokyo	B
Ako[35]	125	R	Duke	Pos	46	70	6	45		6		25	0	16	80-90	Japan	B
Allal[36]	101	R	Other	N/A	56	70	18	11		9	17			4	66-82	France	C
Al-Tawfiq[37]	54	R	Mod Duke	Def	60	68	43	17	7	22		29		19	95-08	Saudi Arabia	B
Auger[38]	50	R	Pelletier	Pos	43		6	28			14				69-77	Canada	C
Avanekar[39]	600	P	Mod Duke	NR	63	67	22	32	7	10				34	80-98	USA	B
Bailey[40]	210	R	Hickie	N/A	43	70	14	25			30	31	0	8	62-71	Australia	C
Barrau[41]	170	P	Mod Duke	Def	65	74	13	18	7	10	11		3	41	14-00	France	A
Bennis[42]	157	R	Other	N/A	28	63	4		11		22			4	83-94	Morocco	C
Bishara[43]	252	R	Mod Duke	Pos	62	54	24	32	11	6	25	16	0	23	87-96	Israel	B
Borer[44]	71	R	Duke	Pos	50	55	4	18		18	8	17	1	27	80-94	Israel	B
Bouramoue[45]	32	R	Von Reyn	Pos			19				41				76-89	Congo	B
Bouza[46]	109	P	VReyn/Steckelberg/Duke	Pos	50	73	45	9	14	9	10		36	17	94-96	Spain	B
Braun[47]	261	P	Von Reyn/Duke	Pos	49	69	18	31		10	23	16		28	80-99	Chile	B
Buchholtz[48]	235	P	Mod Duke	Pos	61	70	22		9	18	12	15	6	25	02-05	Denmark	B
Cabell[49]	329	P	Duke	Pos	57	54	40	11	9	10	10		8	27	93-99	USA	B
Casabe[50]	294	P	Duke	Pos	51	70	26	31	2	11	20	24	14	9	92-93	Argentina	B
Cecchii[51]	147	P	Mod Duke	Def			19	18	8		25	14	10	25	00-01	Italy	A
Cetinkaya[52]	189	R	Mod Duke/Von Reyn	NR	36	66	12	8	5	6	50		0	20	74-99	Turkey	B
Chao[53]	88	R	Duke	Pos	41	67	32	17	2	3	20	25	24	8	90-97	Taiwan	B
Chen[54]	58	R	Duke	Def	41	79	21	55	9	3	5				87-98	Taiwan	B
Chen[55]	178	R	Von Reyn	Pos	62		39	27		6				25	79-91	Australia	B
Cheng[56]	101	R	Other	Pos	39		8	53		4	14				79-87	Taiwan	C
Choudhury[57]	190	R	Other	N/A	25	70	17	8	3	2	54	25		1	81-91	India	C
Chu[58]	65	R	Duke	Pos	68	29	31	25	2	5	23	20		23	97-02	NZ	B
Cicalini[59]	151	R	Duke	Def	44	69	42	19	8	7	17		60	11	92-03	Italy	B
Cicalini[59]	132	R	Duke	Def	36	65	44	23	6	5	15		58	12	80-91	Italy	B
Corral[60]	550	R	Mod Duke	Pos	51	73	44	15	12	5	8		41		85-02	Spain	B
Couturier[61]	66	R	Duke/Von Reyn	Def	57	65	26			6	20		5	14	92-98	France	B
Deprele[62]	80	R	Duke	NR	65	70	7	21	6	11	11			10	95-01	France	B
Di Salvo[63]	178	R	Duke	Def	57	75				5	12	11	3	26	93-00	France	B
Durack[64]	204	R	Duke	Def	48	51	37	23	7	7	3	21	28	31	85-92	USA	B
Dwyer[65]	193	R	Von Reyn	Pos	51	65	39	26	5	5	10		9	22	79-92	Australia	B
Dyson[66]	128	R	Other	N/A	53	70	11	38	15	13	5	17	0	39	87-96	UK	C
Fedorova[67]	112	R	Duke	NR	51	65	23			4	41	26	15	3	00-07	Russia	B
Fefer[68]	108	R	Duke/Von Reyn	Pos	57	56	13	31	7	11	18	11		31	90-99	Israel	B
Ferreiros[69]	390	P	Mod Duke	Def	59	70	28	25	6	10	11	25	4	16	01-02	Argentina	A
Finland[70]	78	R	Other	N/A			31	18		9					60-64	USA	C
Garvey[71]	165	R	Other	N/A		56	14	32	6		16	30	7	20	68-73	USA	C
Gergaud[72]	53	R	Von Reyn	Pos	66	66	11	19	4	11	15		13	17	83-90	France	B
Giannitsioti[73]	195	P	Mod Duke	Pos	64	65	17	21	10	20	10	20	7	22	00-04	Greece	B
Gossius[74]	46	R	Von Reyn	Pos			37	20	13	4	20			17	72-81	Norway	B
Gotsman[75]	100	R	Duke	Def	55	55	17	22	5	4	12	8	1	24	91-00	Israel	B
Gracey[76]	49	R	Other	N/A	39		14	49							60-65	Australia	C
Haddy[77]	23	R	Cherubin	N/A			39				0				73-79	USA	C
Haddy[77]	43	R	Cherubin	N/A			7				16				64-73	USA	C
Hammami[78]	72	R	Duke	Def	32	56	18	17	6	1				67	97-00	Tunisia	B
Heiro[79]	95	R	Duke	Pos	55	70	33	20	8	6	18		26	16	00-04	Finland	B
Heiro[79]	125	R	Duke	Pos	58	72	24	22	10	6	29	11	5	20	90-99	Finland	B
Heiro[79]	106	R	Duke	Pos	51	56	13	19	9	14	34		0	26	80-89	Finland	B
Heper[80]	74	R	Other	N/A	25	66	14	19	15	10		18		14	95-99	Turkey	C
Hermida Amej[81]	87	R	Mod Duke	Def	55	76	44	18	7	8	2		30		89-03	Spain	B
Hill[82]	203	P	Mod Duke	Def	67	60	31	12	11	17	11		1	34	00-04	Belgium	A
Hricak[83]	190	P	Duke/Von Reyn	Pos				16		6	33				02-06	Slovakia	B
Hricak[83]	339	P	Duke/Von Reyn	Pos				15		7	41				91-01	Slovakia	B
Hricak[83]	75	P	Duke/Von Reyn	Pos				15		12	11		0		84-90	Slovakia	B
Hsu[84]	315	R	Mod Duke	Pos	51	59		22	6	5	20		4	8	95-03	Taiwan	B
Huang[85]	72	R	Mod Duke	Pos	58	50	35		4	3	25	30	19	4	03-06	Taiwan	B
Husebye[86]	68	R	Duke	Def	58	69	38	21	4	3	12	34	10	18	88-94	Norway	B
Jaffe[87]	70	R	Other	N/A	47	57	34	26	3	9	10	10	29	16	83-88	USA	C
Jain[88]	247	R	Mod Duke	Pos		71	57	19	5	4	5	15	75	3	96-03	USA	B
Jalal[89]	466	R	Other	N/A	23	59	12	10	2	0	68			1	82-97	India	C
Julander[90]	217	R	Other	N/A			39	23	2	4			27		65-80	Sweden	C
Kanafani[91]	89	R	Mod Duke	Pos	48	64.	20	20	8	3	22	18	0	20	86-01	Lebanon	B
Kazanjian[92]	60	P	Pelletier/Von Reyn	NR	62	57	27	15	10	8	5		28	7	84-89	USA	B
Khanal[93]	46	NR	Duke	Def	26	57	11	13	2			40		2	95-97	India	B
Kim[94]	56	R	Von Reyn	Pos	52	71	14	47	5	4		20	7	23	75-87	USA (HI)	B
King[95]	30	R	Pelletier	Pos	45	60	10	23			17			10	80-82	USA	C
King[95]	13	R	Pelletier	Pos	44	46	8	54			15			0	70-72	USA	C
Kiwan[96]	60	P	Other	N/A	28		12	32						7	85-88	Kuwait	B
Knudsen[97]	51	P	Mod Duke	NR	58	75	25	31	8	16	8	16	6	6	00-01	Denmark	B
Knudsen[97]	121	P	Mod Duke	NR	64	78	25	25	15	15	9	22	3	45	05-06	Denmark	B
Koegelenberg[98]	60	P	Duke	Pos	38	58	2	10	3	2	65		0	167	97-00	South Africa	B
Koga[99]	55	R	Other	N/A			9	22			11	36		20	75-83	Japan	C
Krecki[100]	69	R	Duke	Def	52	59	10			7	41	39		10	92-05	Poland	B
Kurland[101]	154	R	Other	N/A	54	44	27	24	7	7	23	14	4	12	83-92	Sweden	C
Leblebicioglu[102]	112	P	Mod Duke	Pos	45	50	35	29	15	16	16		8	17	00-04	Turkey	B
Lederman[103]	123	R	Mod Von Reyn	NR	42	55	23	34	6	6	6	10	29	10	72-84	USA	B
Leitersdorf[104]	92	R	Pelletier	NR	43		14	37	5		21			33	70-80	Israel	C
Letaief[105]	440	P	Mod Duke	Pos	32	55	12	11	6	4	50	21	0	17	91-00	Tunisia	B
Lien[106]	72	R	Pelletier/Von Reyn	Prob	55	58	21	33	4	4	18		0	10	73-84	Norway	C
Lode[107]	103	P	Other	N/A	46	54	12			12			3	9	71-81	Germany	B
Lopez-Dupla[108]	120	R	Mod Duke	Pos	51	68	33	24	10	6	13	19	25	6	90-04	Spain	B
Lou[109]	120	R	Duke	NR	43	66					37				97-07	China	B
Loupa[110]	101	P	Duke	Pos	55	70	22	19	16	3	18		3	31	97-00	Greece	B
Lowes[111]	60	R	Other	N/A		60	7	52	2	12	25		2	3	66-75	UK	C
Lupis[112]	36	R	Mod Duke	Def	54	56	8			11	58		8		03-06	Italy	B
Manzano[113]	586	P	Duke	NR			18	16	16	8	14		5	39	95-05	Spain	B
Math[114]	104	P	Mod Duke	Def	24	71	7	7		5	66	26	0	23	04-06	India	A
Meirino[115]	131	R	Other	N/A	35	51	14	45				28	2	33	90-94	Mexico	C
Mesa[116]	145	R	Other	N/A	42	62	25	20	12		24		24	37	78-87	Spain	C
Mills[117]	144	R	Vogler	N/A	45	66	24	36	1	7	17	30	19		63-71	USA	C
Morelli[118]	13	R	Other	N/A	58	62	0	46	0	23	31	8	8	31	91-93	Italy	C
Mouly[119]	90	R	Duke	Pos	60	67	31	8	13	8	12	20	8	25	97-98	France	B
Murdoch[120]	2781	P	Mod Duke	Def	58	68	31	17	11	10	10	18	10	22	00-05	Multicenter	A
Nadji[121]	310	P	Duke	Def	60	74	23		7	10	19			13	90-03	France	A
Nakamura[122]	93	R	Other	N/A	34	58	8	47			24				76-81	Japan	C
Nashmi[123]	47	R	Mod Duke	Def	32	59	24	11	17	13	26	9	4	21	93-03	S. Arabia	B
Netzer[124]	125	R	Duke	Pos			24		10	35	7	14	13	17	88-95	Switzerland	B
Netzer[124]	87	R	Duke	Pos			21	26	12		9	16	7	17	80-87	Switzerland	B
Nigro[125]	18	R	Duke	Pos	43	72	22	11			28			33	96-99	Italy	C
Nihoyannopoulos[126]	109	R	Other	N/A	43	55	16				14			29	68-82	UK	C
Nunes[127]	62	P	Mod Duke	Pos	45	63	21		10	10	36	31		31	01-08	Brazil	B
Okada[128]	28	R	Duke	Def	45	46	0	50	0	11	7			7	87-97	Japan	B
Olaison[129]	161	P	Duke/Von Reyn	NR	60	50	27	23	5	7	22	10	4	16	84-88	Sweden	B
Olds[130]	43	R	Von Reyn	Pos		61	17	17	5		7			24	85-89	USA	B
Ordonez[131]	85	P	Duke	Def	43	71	22			6	28		32	24	92-96	Spain	A
Pachirat[132]	160	R	Duke	Pos	39	66	16	23		6	38	25	8	5	90-99	Thailand	B
Pazdernik[133]	117	R	Mod Duke	Def	60	73	31	9	7	13	18	19	1	18	98-06	Czech Rep	B
Peat[134]	78	R	Von Reyn	Pos	50	54	21	53	6	1		24		21	76-86	NZ	B
Pelletier[135]	125	R	Pelletier	Pos	43	77	30		5	10	10		15	10	63-72	USA	C
Proenca[136]	65	R	Duke	Def			54	3	15	2	23		72	2	88-98	Portugal	B
Quenzer[137]	72	R	Other	N/A			10	26	6	7	24			33	69-72	USA	C
Roca[138]	54	R	Duke	NR	62	61	20	15	13	6	19		20	22	99-04	Spain	B
Romero-Vivas[139]	100	R	Von Reyn		40		23	30			8		7	24	77-82	Spain	C
Rostagno[140]	86	P	Duke	NR	59	65	20	5	13		27	12		35	03-06	Italy	B
Ruiz[141]	168	R	Duke	Pos	38	64	27	16	4	5	39		21	13	92-97	Brazil	B
Sanabria[142]	112	R	Other	N/A			40				7	13	12	5	81-86	USA	C
Sandre[143]	135	R	Duke/Von Reyn	Pos		65	27	4	10	6	10		11	31	85-93	Canada	B
Sarli-Issa[144]	703	P	Pelletier	N/A	36	66	19			7	11		6	31	78-98	Brazil	B
Seibaek[145]	69	R	Other	N/A	51	70	13				12		6	29	83-92	Denmark	C
Sekido[146]	38	R	Duke	Def	43	66	13	34	8		32	16		3	86-96	Japan	B
Shinebourne[147]	63	R	Other	N/A		69	3	40		10	11				56-65	UK	C
Shively [148]	16	P	Other	N/A			38	19		6			23	18	88-89	USA	C
Siddiq[149]	182	P	Other	N/A	46	63	57	21	3	7	4	9	67	9	90-93	USA	B
Singhman[150]	101	R	Other	N/A		59	14	43			24	23		1	68-77	Malaysia	C
Slipczuk	261	R	Mod Duke	Pos	60	49	48	10	7	20	4	25	15	11	00-10	USA	B
Strate[151]	30	R	Other	N/A	51	33	20	37	7	10	13		0	10	75-84	Denmark	C
Sucu[152]	72	R	Mod Duke	Def	45	57	17	17	10	4	36	15		29	04-07	Turkey	B
Svanbom[153]	41	P	Other	Prob	53		22	32	2	7	5				67-71	Sweden	B
Sy[154]	273	R	Mod Duke	Pos	55	68	43	19	4	8	15	23	19	20	96-06	Australia	B
Tariq[155]	66	R	Mod Duke	Pos	24	67	5	18	8	2	48	27	2	8	97-01	Pakinstan	B
Terpenning[156]	154	R	Von Reyn	Prob			36	26	9	10	3		22	18	76-85	USA	B
Thalme[157]	192	R	Duke	Pos	52	55	36	21	10	10	14	9	31	15	94-00	Sweden	B
Thornton[158]	139	R	Other	N/A	41	61	24	36	1	13			3	13	69-79	USA	C
Tornos[159]	104	P	Mod Duke	Pos	57	70	33	13		14	14		8	26	01	Europe	B
Tran[160]	136	R	Duke	Pos	54	61	24	28	9	12	18	15	13	21	98-00	Denmark	B
Tugcu[161]	68	R	Mod Duke	Pos	51	59	28	13	13	2	21	25	0	56	97-07	Turkey	B
Venezio[162]	40	R	Other	N/A			15	35	3					15	72-80	USA	C
Verheul[163]	141	R	Von Reyn	Pos	45	74	18	68							66-91	Netherlands	B
Vlessis[164]	140	R	Von Reyn	Pos	57	65	21		5	9			11	22	82-92	USA	B
Von Reyn[165]	104	R	Von Reyn	Pos		51	25	34	3	7	5		4	21	70-77	USA	B
Wang[166]	70	R	Duke	x	36	54		33		7	40			11	88-00	China	C
Watanakukakorn[167]	210	R	Other	N/A	65	55	47	14		5			16	14	80-90	USA	C
Wells[168]	102	R	Von Reyn	NR	52	64	27	43	3	4	5	27	4	9	79-86	NZ	B
Welsby[169]	91	R	Other	N/A	53	65	4	18	1		11				59-74	UK	C
Weng[170]	109	R	Duke	Pos	38	73	15	22	1	2	49		5	9	84-94	China	B
Werner[171]	106	R	Duke	Def	59		18	28	14	17	13	20	5	26	89-93	Germany	B
Witchitz[172]	228	NR	Von Reyn	Prob			36				9				81-88	France	B
Witchitz[172]	257	NR	Von Reyn	Prob			30			13	9		9	22	73-80	France	B
Wong[173]	57	R	Mod Duke	Pos	66	77	28		7	10	12			28	02-07	NZ	B
Zamorano[174]	151	NR	Duke	Def	51	66	31	13			21		27	33	91-03	Spain	B

Frequency distribution for pathogens, male, in-hospital mortality and, IVDA and prosthetic valve are expressed as percentage of total. De=Design. Cert= Diagnosis certainty. SA= Staphylococcus aureus. SV Strepcococcus viridans. CNS= Coagulase-negative Staphylococcus. Ent= Enterococci. CN= Culture negative. Mort= In-hospital mortality. IVDA= Intravenous drug abuse IE. Prosthetic= Prosthetic valve IE. P= Prospective. R=Retrospective. Def= Definite. Pos= Possible. Prob= Probable. NR= Not reported. N/A= Not applicable.

**Table 2:** Characteristics of population-based studies included.

**Author**	**N**	**De**	**Definition**	**Cert**	**Incidence**	**Case find**	**Age**	**Male**	**SA**	**SV**	**CNS**	**Ent**	**Cn**	**Mort**	**IVDA**	**Prosth**	**Year**	**Country**	**Quality**
Benes[175]	134	P	Mod Duke	Pos	3.4cases/100K/year	MD report hospital	69	60	30	13	8	8	34		8	8	07-08	Czech Rep	B
Benn[176]	62	R	Von Reyn	NR	27 cases/millon/year	D/c statistics	55	58	34	21	5	15	8		5	13	84-93	Denmark	B
Correa de Sa[177]	40	P	Mod Duke	Pos	5.0 to 7.9 cases/100K/year	Registry	71	50	30	30	20	8	5			18	01-06	USA	B
Delahaye[178]	401	P	Von Reyn	Pos	22.4cases/millon/year	Questionnaire	56	64	18	27	5	10	9	21	5	22	90-91	France	B
Goulet[179]	288	P	Von Reyn	Pos	18cases/millon/year	Survey	50		12	37	6	20	10			15	82-83	France	B
Griffin[180]	37	R	Von Reyn	Pos	3.9 cases/millon/year	Registry			33	35		6	3				70-81	USA	C
Griffin[180]	21	R	Von Reyn	Pos	3.3 cases/millon/year	Registry			38	43			10			0	60-69	USA	C
Hoen[181]	390	P	Duke	Def	31 cases/million/year	Survey	60	71	23	16	6	7	5	16	6	16	99	France	A
Hogevik[182]	127	P,R	Mod Von Reyn	Pos	6.2 cases/100K/year	MD report hospital	69	36	31	22	6	5	9	23	7	15	84-88	Sweden	B
King[183]	75	P	Pelletier/Von Reyn	Pos	1.7 cases/100K/year	MD report hospital	48	56	35	25	4	9	3		17	19	85-86	USA	B
Nakatani[184]	848	R	Other	N/A	NR	Survey	55	61	17	32	9	7				12	00-01	Japan	C
Schnurr[185]	70	R	Other	N/A	NR	Registry and hospitand records		61	20	45	7	7	6				73-76	Scotland	C
Scudeller[186]	254	P	Mod Duke	Pos	4.21 cases/100K/year	MD report hospital	67	67	18	17		19	19		2	32	04-08	Italy	B
Smith[187]	78	R	Other	NA	16 cases/million/year	Registry		56	18	24			20				69-72	Scotland	C
Steckelberg[22]	68	NR	Mod Von Reyn	NR	4.2 cases/100K/year	Registry and hospital records			29	40	7	3	12	28	3	35	70-87	USA	B
Tleyjeh[16]	48	P	Mod Duke	Pos	6.3-6.5 cases/100K/year	Registry and hospital records	64	71	29	42	4	8	0		6	25	90-00	USA	B
Tleyjeh[16]	34	P	Mod Duke	Pos	5.0-7.0 cases/100K/year	Registry and hospital records	57	71	20	47	15	6	0			29	80-90	USA	B
Tleyjeh[16]	25	P	Mod Duke	Pos	5.3 - 6.0 cases/100K/year	Registry and hospital records	61	80	28	44	0	0	4			4	70-79	USA	B
Van der Meer[188]	406	P	Von Reyn	Pos	15 cases/million/year	Survey	52	66	20	40		5	1	20	7	20	86-88	Netherla	B
Whitby[189]	71	R	Von Reyn	NR	NR	Registry, MD report and medical records	51	68	13	42	4	9	17				76-81	UK	B

Frequency distribution for pathogens is expressed as percentage of total. De=Design. Cert= Diagnosis certainty. SA= Staphylococcus aureus. SV Strepcococcus viridans. CNS= Coagulase-negative Staphylococcus. Ent= Enterococci. CN= Culture negative. Mort= In-hospital mortality. IVDA= Intravenous drug abuse IE. Prosth= Prosthetic valve IE. P= Prospective. R=Retrospective. Def= Definite. Pos= Possible. NR= Not reported. N/A= Not applicable.

### Hospital-based Studies

**Figure 2:**
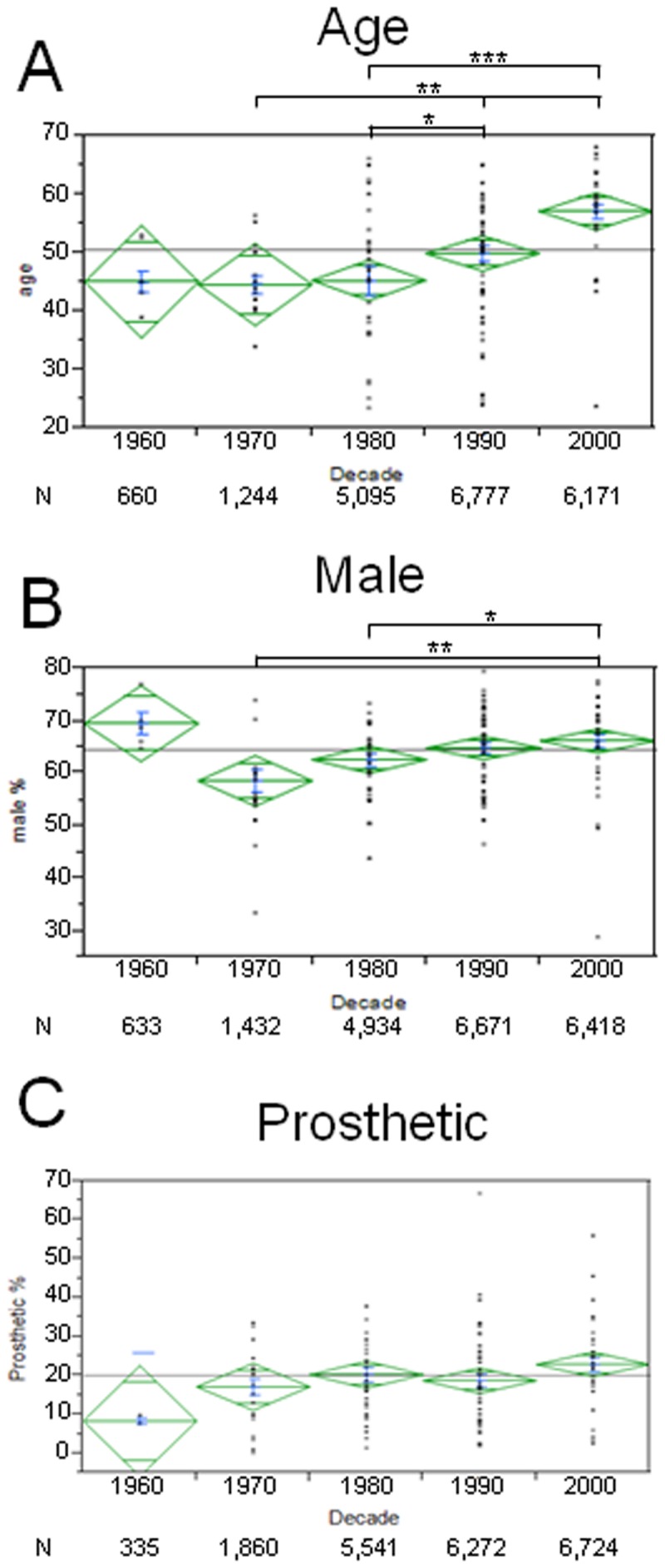
Epidemiology of Infective Endocarditis. Figure shows age (A), male percentage (B) or prosthetic valve IE (C) of patients in each decade (mean in green, centerline of diamond) and variance (as size of diamond) plus standard deviation (blue). Each dot in column represents a particular study mean. N below decades represents total number of patients in each decade. A) IE patients are older in the last two decades. B) Male to female ration increased in the last decade. C) No significant changes were found on prosthetic valve IE. However a trend towards an increase can be seen. *= p<0.05; **=p<0.01; ***=p<0.001.

Among hospital-based studies, IE epidemiology changed over the last 5 decades (Figure 2). Patients were significantly older (Figure 2A; 1980s: mean age 45.3, CI 40.2- 50.5 vs 2000s: mean age 57.2, CI 54.7- 59.7, p<0.001), and more were men (Figure 2B; 1970s: 58.6%, CI 54.3- 63.0 vs 2000s: 66.3%, CI 63.6- 69.0, p<0.01). The percentage of IE cases occurring on prosthetic valves increased over time though with borderline statistical significance (Figure 2C; 1960s: 8.4%, CI -3.8- 20.5 vs 2000s: 22.9%, CI 19.1 - 26.7, p=0.05). 

**Figure 3:**
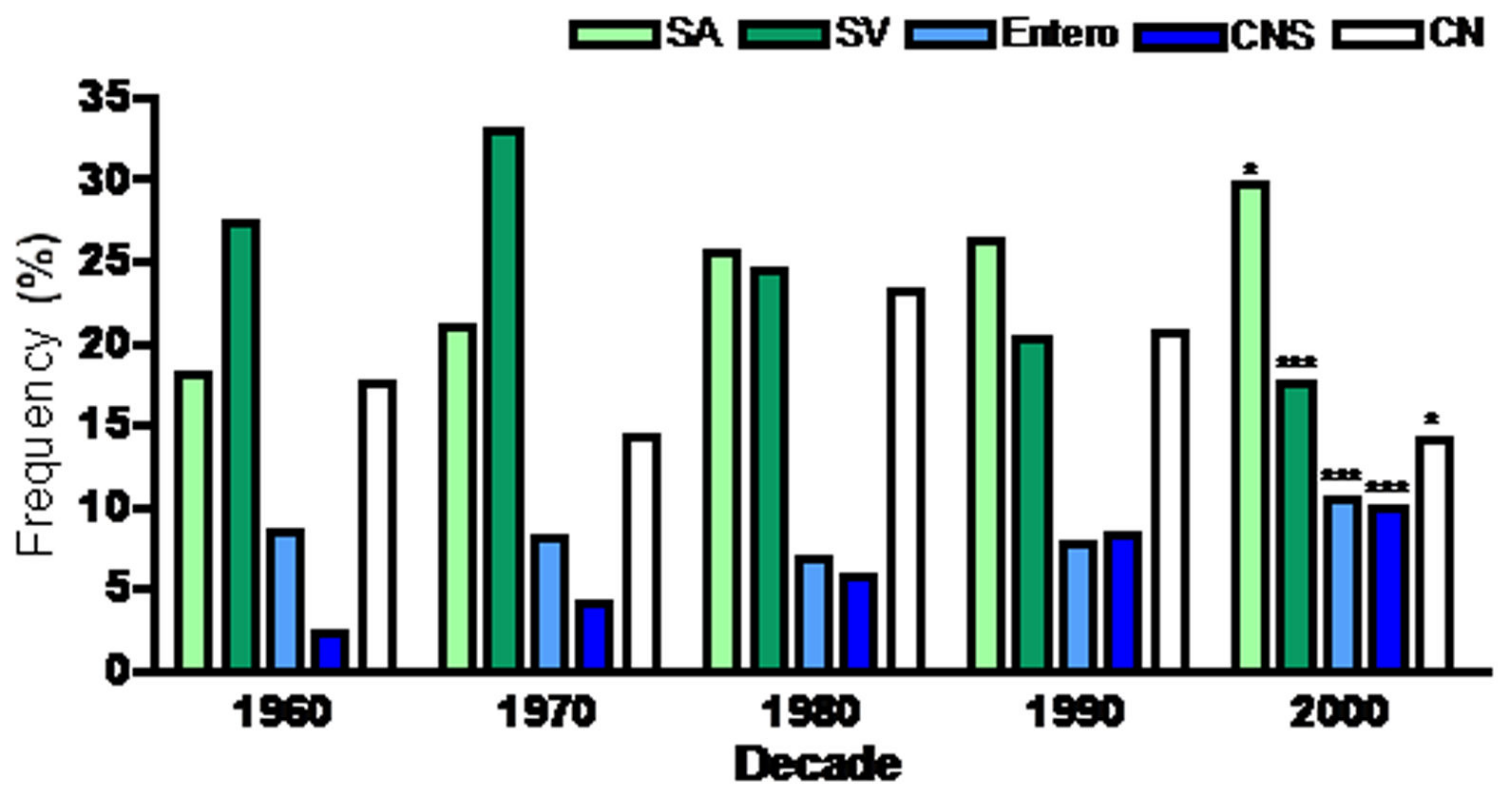
Summary of Worldwide Microbiology of Infective Endocarditis. Bars represent percentage of *Staphylococcus* *aureus* (SA) (light green), Streptococcus viridans (SV, dark green), enterococci (Entero, light blue), coagulase-negative staphylococcus (CNS, dark blue), and Culture negative (CN, white) endocarditis in each decade. *= p<0.05; **=p<0.01; ***=p<0.001.

**Figure 4:**
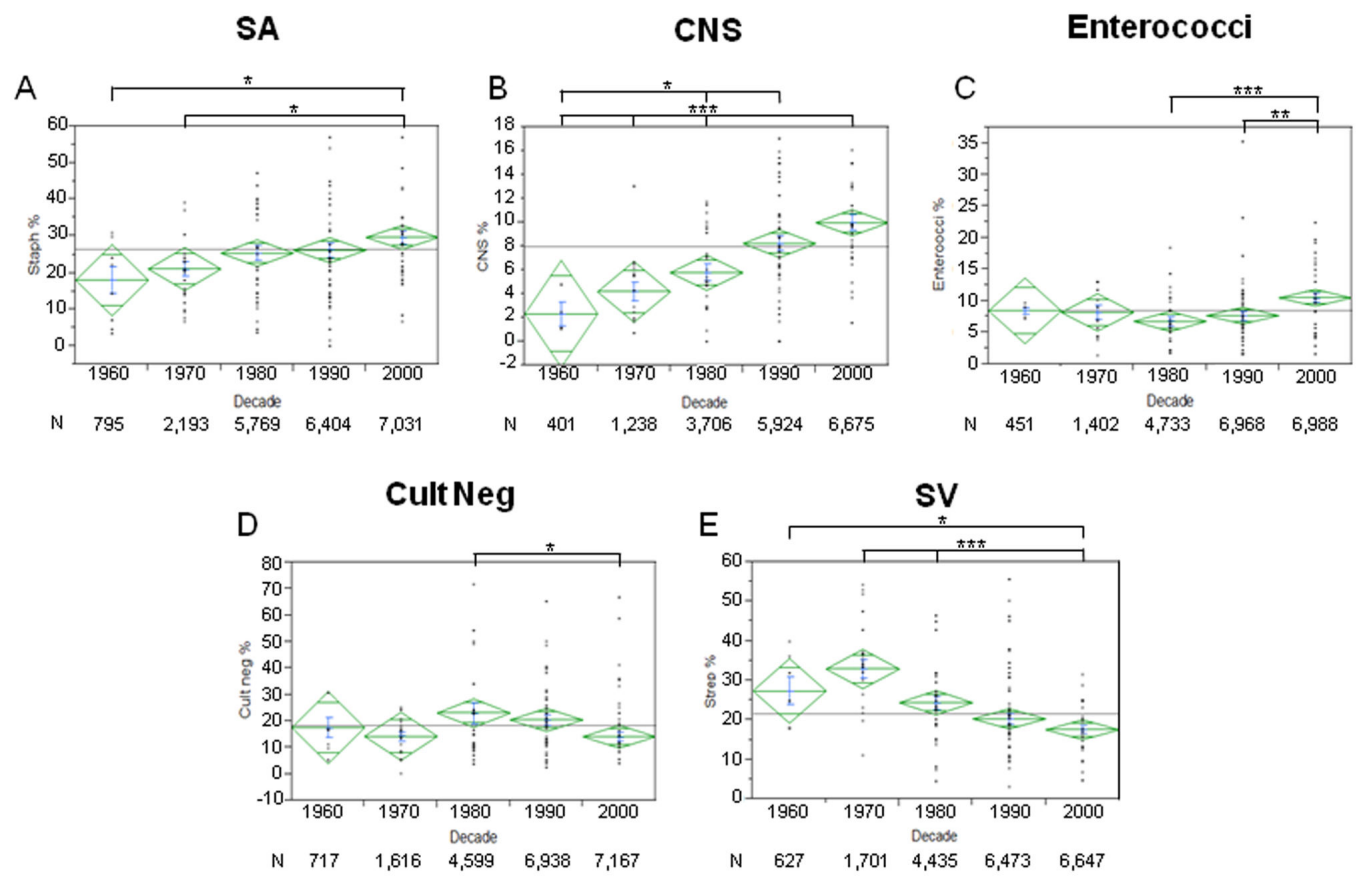
Microbiology of Infective Endocarditis. Figure shows percentage of *Staphylococcus* *aureus* (SA) IE (A), coagulase-negative staphylococcus (CNS, B), enterococci (C), Culture negative (D) and Streptococcus viridans (SV, E) of patients in each decade (mean in green, centerline of diamond) and variance (as size of diamond) plus standard deviation (blue). Each dot in column represents a particular study mean. N below decades represents total number of patients in each decade. A) SA increased in the last decade. B) CNS increased over time. C) enteroccoci increased in the last decade. D) Culture negative endocarditis decreased in the last decade. E) SV decreased over time. *= p<0.05; **=p<0.01; ***=p<0.001.

Changes in microbiology percentage over time are summarized in Figure 3 and shown in e- Figure 1 for individual organisms. There were significant increases in frequency distribution of *Staphylococcus aureus* (SA, Figure 4A) IE (1960s: 18.1% CI 9.4- 26.7 vs 2000s: 29.7%, CI 26.2- 33.3, p<0.05) and coagulase-negative staphylococcus (CNS, Figure 4B) IE (1960s: 2.4%, CI 0.8-5.5 vs 2000s: 10.0%, CI 8.6-11.3, p<0.01). Enterococcal IE percentage increased significantly over the last decade (Figure 4C, 1980s: 6.8%, CI 5.4- 8.2 vs 2000s: 10.5%, CI 8.9- 12.1, p<0.001) while culture negative IE decreased in that time period (Figure 4D, 1980s: 23.1%, CI 15.0- 31.3 vs 2000s: 14.2% CI 9.9- 18.2; p=0.01). Streptococcus viridans (SV) IE markedly decreased in percentage over time span of the study (Figure 4E, 1960s: 27.4%, CI 18.4-36.4 vs 2000s: 17.6%, CI 15.7-19.5, p<0.05). 

**Figure 5:**
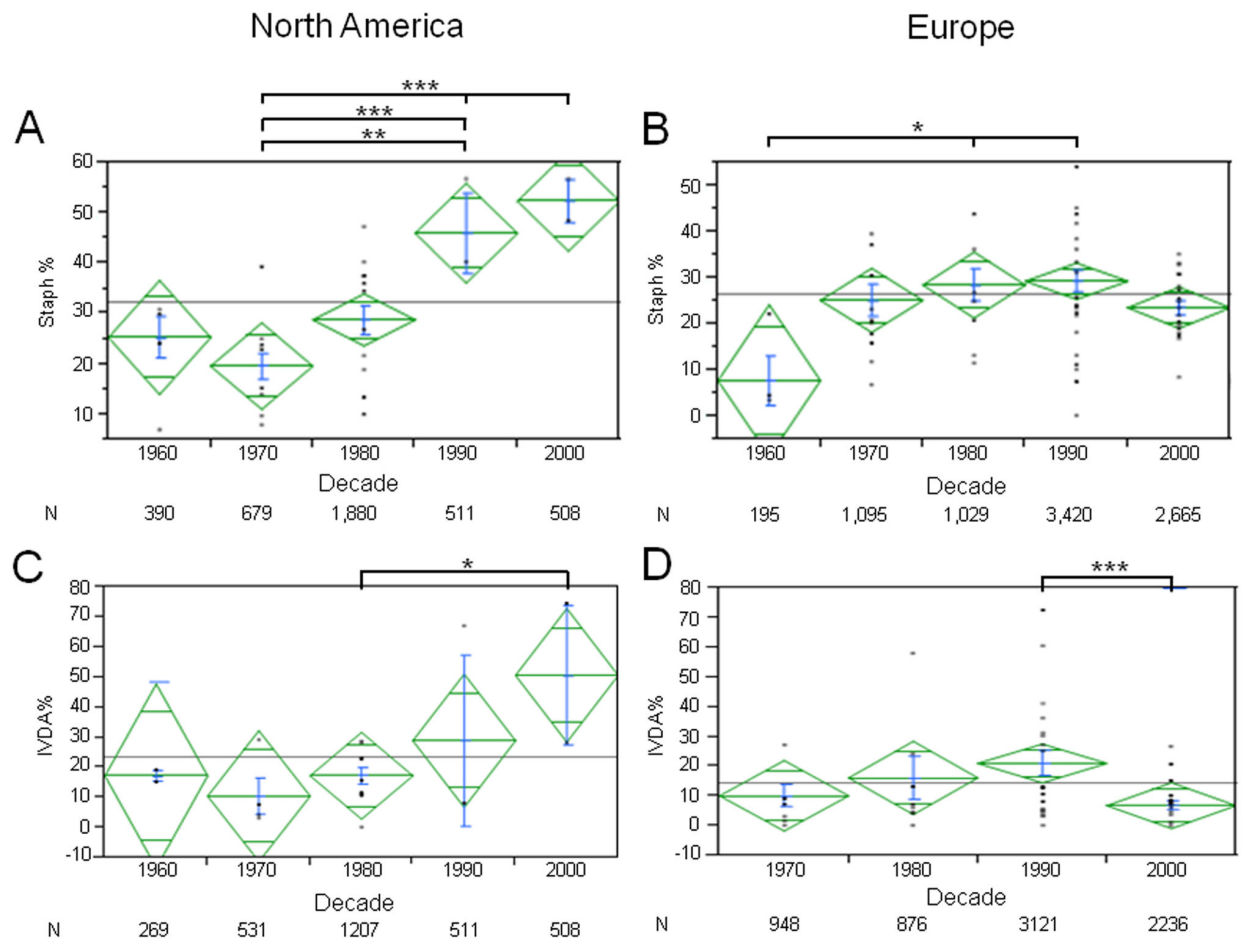
Regional Differences for Staphylococcus Aureus and Intravenous Drug Abuse. Figure shows percentage of *Staphylococcus* *aureus* (Staph, SA) IE in North America (A) or Europe (B) and intravenous drug abuse related IE in North America (C) and Europe (D), of patients in each decade (mean in green, centerline of diamond) and variance (as size of diamond) plus standard deviation (blue). N below decades represents total number of patients in each decade. A) SA increased markedly over last half century in North America B) No changes in SA were found in Europe. C) IVDA related IE frequency increased in North America. D) IVDA related IE percentage decreased in Europe in the last decade. *= p<0.05; **=p<0.01; ***=p<0.001.

Subgroup analyses, by continent, were performed. The increase in overall SA frequency was driven by an increase in North America (Figure 5A; 1960s: 25.3%, CI 13.9- 36.6 vs 2000s: 52.4%, CI 42.4- 62.3, p=0.001). SA percentage in Europe remained stable over the last 4 decades (Figure 5B; 1970s: 25.1%, CI 18.2- 32.1 vs 2000s: 23.5%, CI 19.1- 28.0, p=0.70). No significant differences were found in SA IE frequency in Asia, Africa, Latin America, or Oceania. 

 Counterbalancing the increase in SA IE percentage in North America was a decrease in SV IE frequency (1970s: 33.5%, CI 25.8- 41.3 vs 2000s: 14.4%, CI 5.6- 23.2, p<0.01). SV IE frequency distribution also decreased significantly in Asia (1970s: 41.5%, CI 28.7- 54.4 vs 2000s: 10.1%, CI -8.9- 29.2, p<0.01) while in Europe there was a decrease that did not reach statistical significance (p=0.06). No significant changes were seen in Latin America and Oceania (p=0.9, p=0.32, respectively). Insufficient data were available from Africa for separate analysis. 

Subgroup analyses for changes in IVDA IE percentage are shown in Figure 5. No significant changes were seen on a global basis. However, a significant increase in IVDA related IE frequency distribution was observed in North America in the last decade (Figure 5C; 1980s: 17.3%, CI 10.7- 23.9 vs 2000s: 50.7%, CI 28.5- 73.0, p<0.05). Conversely, we observed a significant decrease in IVDA related IE percentage in Europe in the last decade (Figure 5D; 1990s: 21.1%, CI 12.3- 29.8 vs 2000s: 6.8%, CI 3.5- 10.2, p<0.01). We found a positive correlation between SA IE and IVDA. Interestingly, this correlation lost strength in the last decade (1990s rs=0.82, p=0.001 vs 2000s rs=0.40 p=0.05; 1990s vs 2000s, Fisher r-to-z transformation, p<0.001). We further analyzed the studies that reported microbiology for the IVDA IE group. Twenty-five studies and 1288 patients were included in this sub-analysis. No significant temporal trends in IVDA IE microbiology were found. In this subset of patients, SA represented the main pathogen (1970s: 69.89 CI 31.40- 108.38, 1980s: 72.72 CI 57.98- 93.45, 1990s: 61.78 CI 47.87- 75.68 and 2000s: 65.99 CI 55.12- 76.86) and SV represented a small percentage of cases (1970s: 16.66 CI 3.87- 29.45, 1980s: 8.87 CI 2.79- 14.95, 1990s: 7.37 CI 3.57- 11.19, 2000s: 10.01 CI 7.21- 12.81).

**Figure 6:**
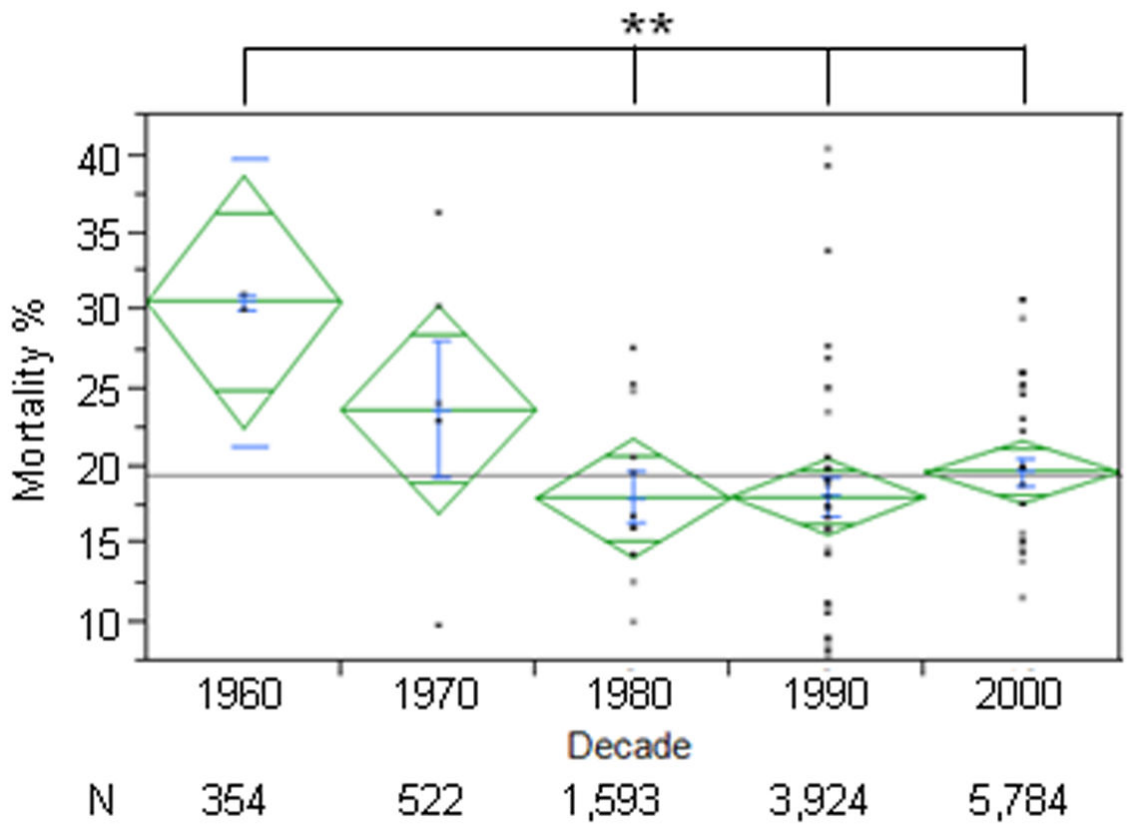
In-Hospital Mortality of Infectious Endocarditis. Figure shows percentage of in-hospital mortality of Infectious Endocarditis in each decade (mean in green, centerline of diamond) and variance (as size of diamond) plus standard deviation (blue). Each dot in column represents a particular study mean. N below decades represents total number of patients in each decade. In-hospital mortality decreased after the 1960s and remained stable thereafter. ∗∗=p<0.01.

In-hospital mortality rate due to IE decreased following the 1960s and remained stable thereafter (Figure 6; 1960s: 30.6%, CI 24.4- 36.8 vs 2000s: 19.7%, CI 17.8- 21.6, p=0.01). On subgroup analysis by continent, no regional differences were observed. 

### Population-based Studies

Among population-based studies, no significant trends were observed regarding IE microbiology as shown in Table 3 (SA p=0.82; SV p=0.14; enterococci p=0.33). These studies included populations in the US and Europe primarily. Only one study was from Asia. Of note, the majority of data from the US is from Olmstead County, Minnesota. 

**Table 3:** Microbiology of Infective Endocarditis from Population-based Studies.

	**1960s**	**1970s**	**1980s**	**1990s**	**2000s**	**p**
**SA**	**38 % [-1%-77%]**	**22% [12%-31%]**	**21% [15%-26%]**	**21% [14%-27%]**	**19% [13%-24%]**	**0.82**
**SV**	**43% [-9%-95%]**	**37% [24%-50%]**	**34% [27%-42%]**	**23% [14%-31%]**	**27% [19%-33%]**	**0.14**
**Entero**		**56% [-3%-14%]**	**13% [8%-19%]**	**8% [3%-13%]**	**9% [5%-13%]**	**0.33**

Among population-based studies, no significant changes were observed regarding infectious endocarditis microbiology incidence over last five decades. Note that percentages are weighted by size and therefore sum of each decade may exceed 100%.

### 2000s data from Einstein Medical Center, Philadelphia, PA, US

We identified a total of 261 cases from 2000 to 2010 (Table 4). Mean age was 59 and 49% were male. In-hospital mortality rate was 25⋅3%. Prosthetic IE represented 10.7% and IVDA 14.6%. SA was the primary IE microorganism seen causing 48.3% of IE [25.3% Methicillin-sensitive *Staphylococcus aureus* (MSSA) and 23.0% Methicillin-resistant *Staphylococcus aureus* (MRSA)] while CNS was seen in 6.9% of the cases. Enterococcus was the IE etiology in 19.2% and SV 9.6%. Culture negative IE represented 3.8%. Removal of this data did not modify overall results. 

**Table 4:** Data from Einstein Medical Center from 2000-2010.

Cases	261
Time frame	2000-2010
Age	59.8
Male	49.4%
In-hosp mort	25.3%
Prosthetic	10.7%
IVDA	14.6%
Staph aureus	48.3%
MSSA	25.3%
MRSA	23.0%
Enterococci	19.2%
Strep viridans	9.6%
Coag Neg Staph	6.9%
Culture neg	3.8%

## Discussion

The main finding of this study is that the epidemiology of IE has changed worldwide over the last half century. Furthermore, the observed changes in IE microbiology varied by continent. These findings stemmed from analyses of hospital-based reports. In a separately analyzed smaller group of population-based studies (most of them from the US), no consistent changes in IE microbiology frequency distribution over time were observed. 

Most notably, the global percentage of SA IE has nearly doubled in the last five decades (18% in the 1960s to nearly 30% in the 2000s). When analyzed by continent this increase was largely due to an increased frequency of SA in North America (from 25% in the 1960s to 52% in the 2000s) with no significant change among reports from other continents. This finding has important implications as SA infections are associated with longer length of stay, higher death rates[23], increase hospitalizations[24], and elevated costs[24]. An increase in IVDA IE percentage in North America as compared to Europe may partially explain these changes in SA frequency distribution. However, the number of studies in the last decade in this analysis is small and therefore this finding should be studied further. Other potential contributors include increases in the elderly population[10], increased numbers of chronically-ill patients[25], increased contacts with the health-care system[26,27], and increasing use of intracardiac and vascular devices. Benito and colleagues[27] found a high percentage of health-care associated infections (related to catheters, dialysis, or immunosuppressive therapy) among US patients with native valve IE; SA was the most common organism isolated. Though the present study was not able to specifically track cardiac device implantation, another recent study found an increased prevalence of staphylococcal IE in these patients[28]. In absolute numbers Bor et al, reported an incidence of infective endocarditis in the US close to 40,000 cases/year [29]. Furthermore, at least when measured by ICD codes the total number of SA cases seems to be increasing in the US [15]. In addition, certain subgroups may behave differently; a well-designed population based study found that SA frequency has increased in Europe in patients without previously known valve disease[5]. It is important to clarify that changes in individual countries may not necessarily follow the trends at a continent level. 

The present study also documents a substantial decline in the frequency of SV IE over the last five decades (27.4% in the 1960s to 17.6% in the 2000s). This finding was statistically significant for North America and Asia with a strong trend in Europe. Therefore, it appears that changes in the epidemiology of this organism are more widespread than for SA. 

Paralleling the increase in SA IE frequency, there was also an increase in CNS IE percentage over time. It is known that CNS infections are often related to the use of intravascular catheters and prosthetic vascular grafts[30]. Thus, the rise in CNS IE may well be health-care related. 

Enterococcal IE frequency increased in the last decade of the study. Enterococcal infections typically affect elderly patients and those with prior valvular damage, diabetes mellitus, indwelling catheters, or who are on hemodyalisis[31]. This finding is extremely important given the high prevalence of multidrug resistant enterococci and therefore the implications on treatment options. Lastly, culture negative endocarditis percentage decreased in the last decade. This is likely because of improved laboratory techniques and culture methods. 

Worldwide, the present study found increases in age among IE patients. This has important implications for treatment and use of health care resources as elderly patients have more comorbidities and may be more prone to infection with certain organisms, such as enterococci. Consistent with the general perception[18], the present study found that in-hospital mortality rate of IE remains high with no significant decrease observed since the 1960s. 

A limitation of the present study is the lack of individual patient level data. This data was not available from older studies and including it for only the last decades would have changed one bias for another one, without adding accuracy. Another limitation is that most of the findings come from hospital-based studies, whereas no significant changes were seen in the population based-studies over time. One possible explanation for this is a lack of power (18 population-based studies covering 3,477 patients vs 142 hospital-based studies covering 23,606 patients). Population-studies are also subject to sample bias: the population studied may not truly represent the general population. They can be subject to underreporting, as many times they rely on surveys. Moreover, population-studies of IE in the US are mainly from the Olmstead County, a population that is unlikely to represent the total US population. Hospital-based studies can suffer from referral bias as well, with sicker patients being referred to specialized centers. Thus, these results might not apply to community hospitals. However, Kanafani and colleagues[32] found only a slight difference between referred and non-referred patients, with higher SA IE in the non-referred patients. Thus, had this played a role in the present study, it would have likely decreased SA frequency. Therefore, it is unlikely to explain our findings. Finally, the definition of IE has changed over time, as well as culture quality, which could have caused heterogeneity in the cases included.

## Conclusion

The present study represents the largest systematic review of IE epidemiology to date. Important findings include an increase in staphylococcal IE frequency over the last half-century, particularly in North America, and a worldwide decrease in SV IE percentage. In the last decade SA IE and enterococci IE frequencies have increased while culture negative IE has decreased. Patients with IE are getting older and the male to female ratio is increasing. Mortality has changed little in the last four decades. 

## Supporting Information

Checklist S1 (DOC)
