# Correction: Infective Endocarditis Epidemiology Over Five Decades: A Systematic Review

**DOI:** 10.1371/journal.pone.0111564

**Published:** 2014-10-15

**Authors:** The PLOS ONE Staff

There is an error in the first sentence of the second paragraph of the “Hospital-based Studies” subsection of the Results. The correct sentence is: Changes in microbiology percentage over time are summarized in Figure 3 and shown in Figure 4 for individual organisms.
